# Periostin expression in neoplastic and non-neoplastic diseases of bone and joint

**DOI:** 10.1186/s13569-018-0105-y

**Published:** 2018-09-05

**Authors:** Jennifer M. Brown, Akiro Mantoku, Afsie Sabokbar, Udo Oppermann, A. Bass Hassan, Akiro Kudo, Nick Athanasou

**Affiliations:** 1 Nuffield Department of Orthopaedics, Rheumatology and Musculoskeletal and Sciences, Nuffield Orthopaedic Centre, University of Oxford, Oxford, OX3 7HE UK; 2 Department of Biological Information, Tokyo Institute of Technology, Yokohama, 226-8501 Japan

**Keywords:** Periostin expression, Bone tumours, Tumour progression

## Abstract

**Background:**

Periostin is a matricellular protein that is expressed in bone and joint tissues. To determine the expression of periostin in primary bone tumours and to assess whether it plays a role in tumour progression, we carried out immunohistochemistry and ELISA for periostin in a range of neoplastic and non-neoplastic bone and joint lesions.

**Methods:**

140 formalin-fixed paraffin-embedded sections of bone tumours and tumour-like lesions were stained by an indirect immunoperoxidase technique with a polyclonal anti-periostin antibody. Periostin expression was also assessed in rheumatoid arthritis (RA) and non-inflammatory osteoarthritis (OA) synovium and synovial fluid immunohistochemistry and ELISA respectively.

**Results:**

Periostin was most strongly expressed in osteoid/woven bone of neoplastic and non-neoplastic bone-forming lesions, including osteoblastoma, osteosarcoma, fibrous dysplasia, osteofibrous dysplasia, fracture callus and myositis ossificans, and mineralised chondroid matrix/woven bone in chondroblastoma and clear cell chondrosarcoma. Reactive host bone at the edge of growing tumours, particularly in areas of increased vascularity and fibrosis, also stained strongly for periostin. Vascular elements in RA synovium strongly expressed periostin, and synovial fluid levels of periostin were higher in RA than OA.

**Conclusions:**

In keeping with its known role in modulating the synthesis of collagen and other extracellular matrix proteins in bone, strong periostin expression was noted in benign and malignant lesions forming an osteoid or osteoid-like matrix. Periostin was also noted in other bone tumours and was found in areas of reactive bone and increased vascularity at the edge of growing tumours, consistent with its involvement in tissue remodelling and angiogenesis associated with tumour progression.

## Background

Periostin, a secreted extracellular matrix protein that belongs to the fasciclin family, was originally characterised in osteoblasts and first termed osteoblast-specific factor 2 [1, 2]. Periostin is a matricellular protein that does not have a specific structural role but rather interacts with cell surface receptors, proteases and other molecules that modulate cell adhesion/migration and the fibrillogenesis of collagen and other extracellular matrix (ECM) proteins [3–6]. Periostin has a multi-domain structure in which particular domains bind to many proteins and enzymes that promote ECM protein crosslinking. Periostin is involved in the formation and maintenance of normal bone and teeth tissues and is highly expressed in tissue components that are subject to mechanical stress, such as the periosteum and the periodontal ligament. It has also been observed in other organs and tissues including heart, breast, lung, thyroid, skin, placenta and ovary [3–6].

Periostin is expressed at sites of injury/repair and inflammation [3, 6, 7]. It has been identified in rheumatoid arthritis (RA) and osteoarthritis (OA) joints [8, 9] with a recent study identifying periostin as a key regulator in RA synoviocyte migration/invasion associated with pannus formation [10]. Periostin is also expressed in a number of cancers where it is thought, by various mechanisms, to play a role in tumour progression [3, 6, 11, 12]. Periostin has been identified in a few bone tumours, including fibrous dysplasia and osteosarcoma [13–15], but its expression in other bone neoplasms has not been fully investigated.

In this study we investigated immunophenotypic expression of periostin in a wide range of primary tumours and tumour-like lesions of bone as well as in bone secondaries and metastatic osteosarcomas. Our aims were two-fold: first, to determine whether periostin expression is increased in specific bone tumour types; and second, to examine whether periostin plays a role in tumour progression.

## Methods

### Neoplastic and non-neoplastic tissue samples analysed

Tissue samples from 140 biopsies or surgical resections of bone tumours and tumour-like lesions, were retrieved from the files of the Nuffield Orthopaedic Centre, Histopathology Department, Oxford (Table 1). Criteria for the histological diagnosis of bone and joint lesions investigated in this study were those of the 2013 WHO Classification of Tumours of Soft Tissue and Bone [16]. The tissues were fixed in 10% buffered formalin and, where necessary, decalcified in 5% nitric acid or EDTA. In addition, formalin-fixed paraffin-embedded sections of synovial tissue derived from patients with RA (n = 21) and OA (n = 19) were examined. Samples of normal bone and joint tissues from amputation specimens of individuals with no history or evidence of joint disease or neoplasia were used as controls. Synovial fluid (SF) was also aspirated from the knee joint of nine patients with OA and nine patients with RA. Ethics approval was obtained from the National Research Ethical Committee, and patient consent was acquired prior to the collection of samples.

**Table 1:** Bone tumours/tumour-like lesions analysed in this study

Tissue type	Number analysed
Osteoma	2
Osteoid osteoma	4
Osteoblastoma	6
Osteosarcoma, conventional	20
Osteosarcoma, telangiectatic	2
Osteosarcoma, small cell	2
Osteosarcoma, parosteal	2
Fibrous dysplasia	10
Osteofibrous dysplasia	2
Fracture	2
Myositis ossificans	2
Enchondroma	3
Chondroblastoma	9
Chondromyxoid fibroma	3
Chondrosarcoma, conventional	20
Chondrosarcoma, clear cell	5
Giant cell tumour of bone	8
Aneurysmal bone cyst	4
Solitary bone cyst	2
Non-ossifying fibroma	3
Undifferentiated pleomorphic sarcoma	3
Ewing sarcoma	6
Adamantinoma of long bone	1
Chordoma	2
Plasma cell myeloma	2
Lymphoma	2
Langerhans cell histiocytoma	2
Metastatic breast carcinoma	3
Metastatic melanoma	1

### Immunohistochemistry for periostin

Immunohistochemical staining for periostin was carried out by an indirect immunoperoxidase technique (without any antigen retrieval procedure) using a polyclonal rabbit antiserum against human periostin raised by using peptide DNLDSDIRRGLESNVN (representing aminoacids 143–158 of human periostin) as an immunogen [13, 17]. As in previous studies [17], the antibody dilution was 1:250 and sections of normal skin were employed as a positive control.

### Periostin expression in OA and RA synovial fluid

A quantitative measure of the level of periostin in knee joint RA and OA synovial fluid was determined by ELISA (enzyme-linked immunosorbent assay). The periostin concentration was assessed using a Human periostin/OSF-2 ELISA kit (Adiopo bioscience, Santa Clara, CA, USA). Statistical evaluation was performed using the Mann–Whitney U test with p values less than 0.05 considered as statistically significant.

## Results

### Periostin expression in normal bone and joint tissues

In normal bone there was strong expression of periostin in collagenous fibrous tissue of the periosteum. At points of tendon or ligament insertion into bone, strong periostin staining was also seen within collagenous tissue. There was no staining for periostin in normal lamellar cortical and cancellous bone, and osteocytes, bone lining cells, osteoblasts and osteoclasts did not express periostin. Fatty and hematopoietic marrow was generally negative for periostin but, in some specimens, staining for periostin was noted in small sinusoidal vascular channels within the marrow. In normal joints, the synovium and hyaline articular cartilage did not stain for periostin.

### Periostin expression in tumours and tumour-like lesions of bone and joint

**Fig. 1:**
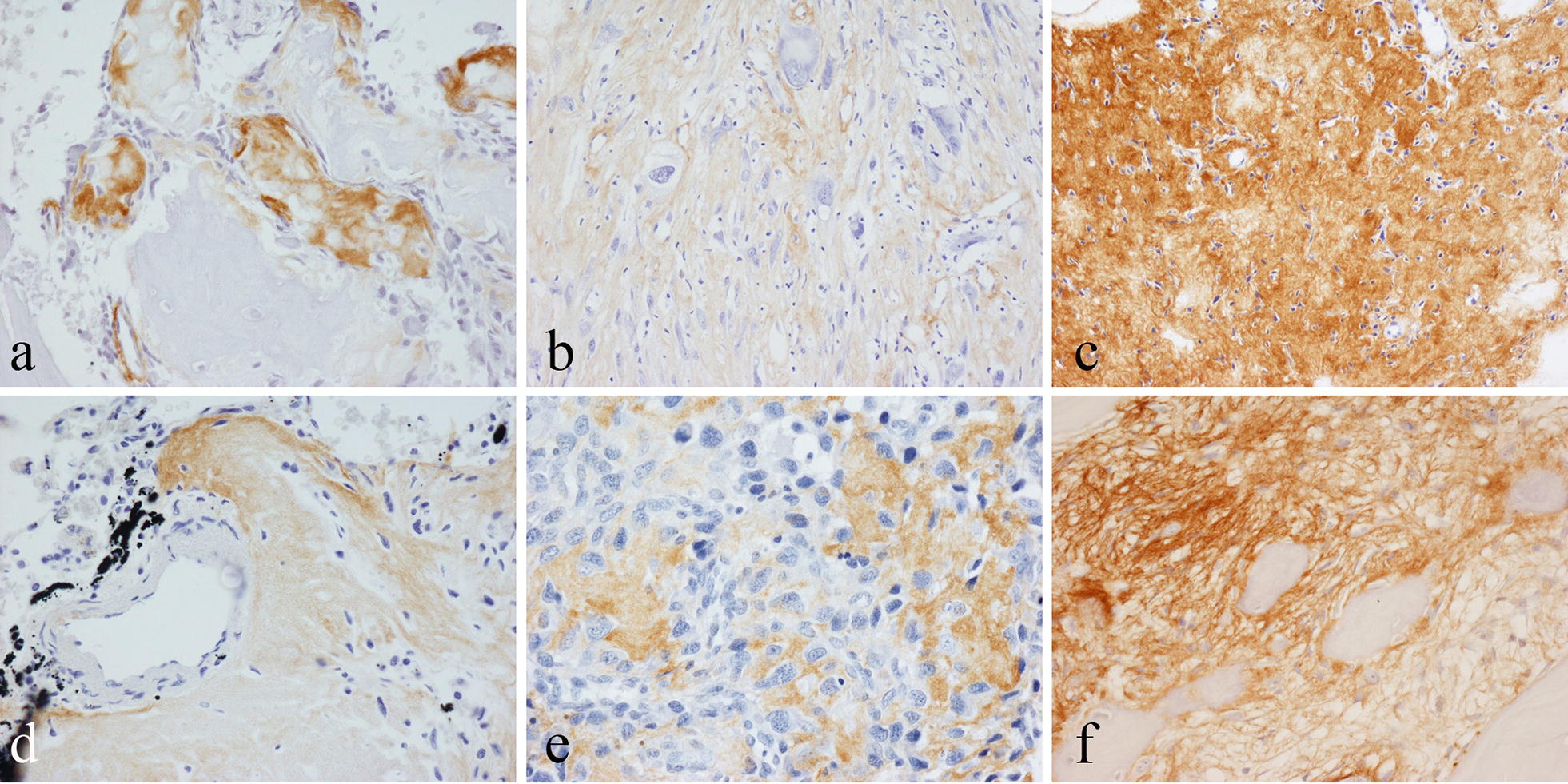
Immunohistochemical staining for periostin in osteoid/newly formed bone of: **a** osteoblastoma; **b** high-grade osteoblastic osteosarcoma; **c** osteoid-rich area of high-grade osteosarcoma; **d** small cell osteosarcoma; **e** osteosarcoma metastasis in lung; **f** fibrous dysplasia

Strong staining for periostin was seen in the osteoid matrix formed by osteoblastic cells in neoplastic and non-neoplastic bone-forming lesions. Periostin staining was noted in the osteoid matrix covering organized reactive bone in fracture callus and myositis ossificans. In osteoblastoma, newly formed osteoid stained strongly for periostin (Fig. 1a); staining for periostin was less pronounced in woven bone and was absent in lamellar bone surrounding the lesion. In osteosarcoma, there was strong staining for periostin in the osteoid matrix formed by malignant cells (Fig. 1b); staining for periostin was seen in all osteosarcomas but the extent of expression was variable with osteoid-rich tumours showing the strongest and most diffuse staining (Fig. 1c). A similar pattern of staining for periostin was noted in lung nodules of metastatic osteosarcoma (Fig. 1d). In small cell osteosarcoma, periostin was expressed in the matrix between tumor cells (Fig. 1e). In chondroblastic and telangiectatic osteosarcomas, cartilage and giant cell components of the tumor were negative. In parosteal osteosarcoma, periostin expression was noted focally in the matrix in areas of tumor cell proliferation. There was strong staining for periostin in fibrous dysplasia and osteofibrous dysplasia, predominantly in the cellular fibrous stroma between bone trabeculae (Fig. 1f).

**Fig. 2:**
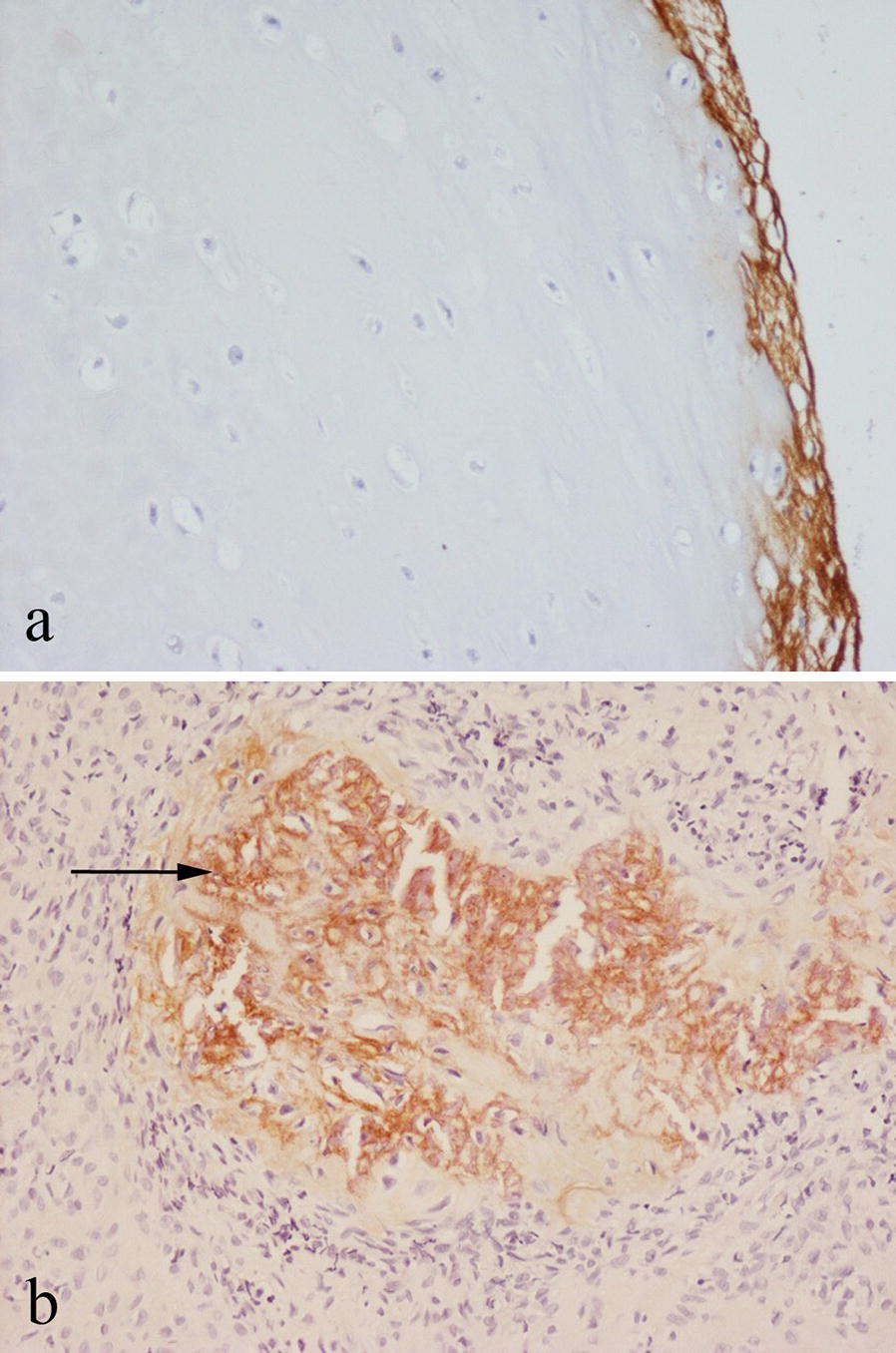
Immunohistochemical staining for periostin in: **a** benign osteochondroma showing staining of the perichondrium covering the (unstained) cartilage; **b** chondroblastoma showing staining in areas containing mineralised chondroid (arrowed)

There was no specific staining of the chondroid matrix or cartilage cells for periostin in enchondroma, osteochondroma, or low/high-grade conventional chondrosarcoma. The dense fibrous perichondrium covering osteochondromas (which is continuous with the periosteum) stained strongly for periostin (Fig. 2a). At the base of growing osteochondromas, there was focal staining of the matrix and thin-walled vessels in areas of enchondral ossification and remodeling of newly formed bone. In chondroblastoma, areas of chondroid matrix, some of which showed evidence of mineralisation, stained focally for periostin (Fig. 2b). There was also focal staining of the matrix around chondroblasts. There was no staining for periostin in chondromyxoid fibroma. In clear cell chondrosarcoma, periostin staining was seen in the mineralized osteoid-like matrix covering woven bone and cartilage formed by vacuolated tumor cells.

**Fig. 3:**
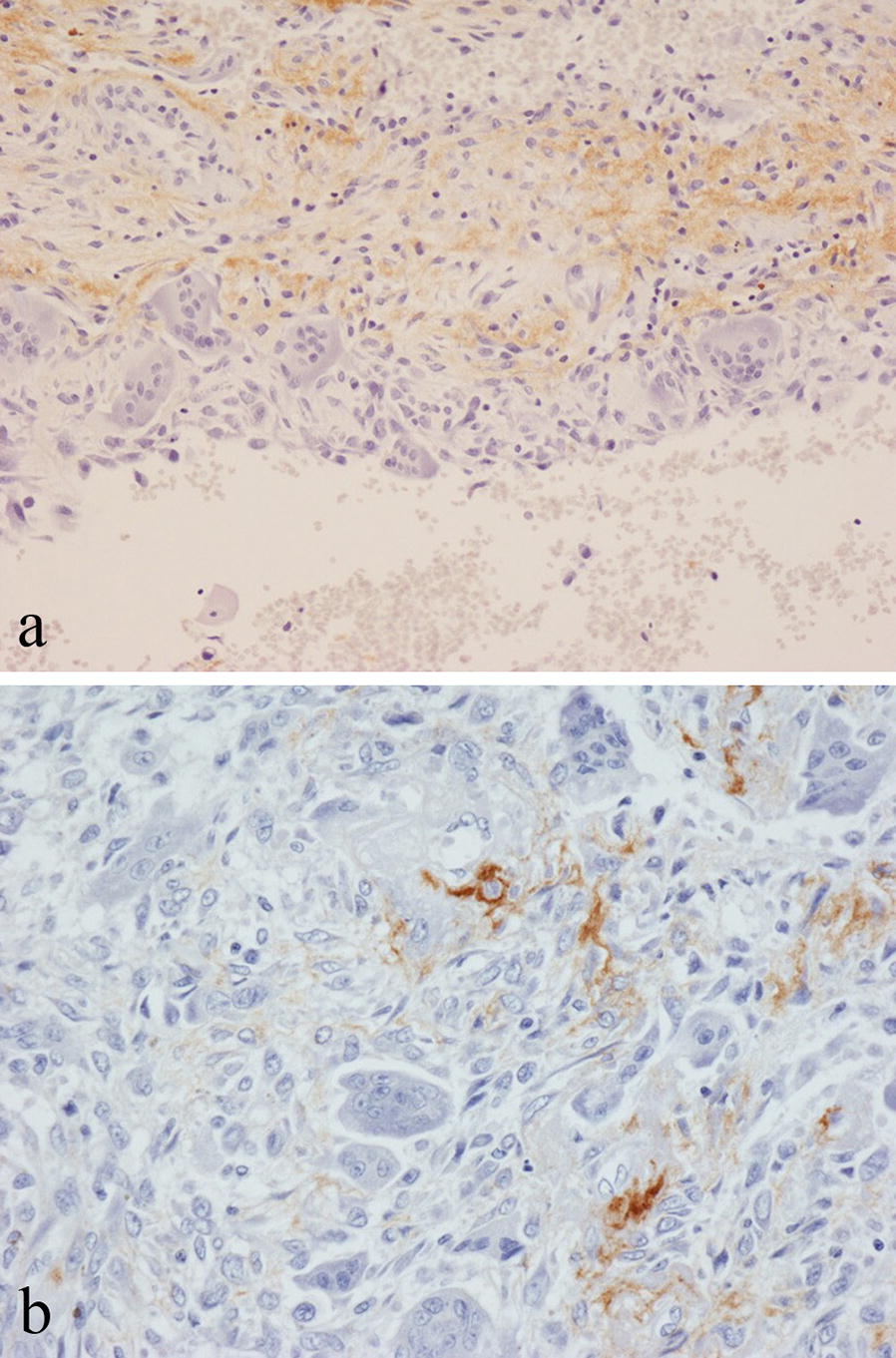
Immunohistochemical staining for periostin in: **a** aneurysmal bone cyst showing matrix staining in fibrous tissue of the cyst wall; **b** giant cell tumour of bone showing matrix staining around mononuclear cells

In aneurysmal bone cyst (ABC) and simple bone cyst, the fibrous stroma and reactive bone within the cyst wall was positive for periostin (Fig. 3a). In giant cell tumour of bone (GCTB), there was focal, occasionally strong staining for periostin in the collagenous connective tissue matrix around mononuclear cells (Fig. 3b). Giant cells in GCTB, chondroblastoma and ABC were negative for periostin. Variable focal staining for periostin was seen in cellular and collagenous connective tissue of other bone tumours, including non-ossifying fibroma and undifferentiated pleomorphic sarcoma. No specific staining for periostin was noted in Langerhans cell histiocytosis, Ewing sarcoma, lymphoma, myeloma, chordoma or adamantinoma.

**Fig. 4:**
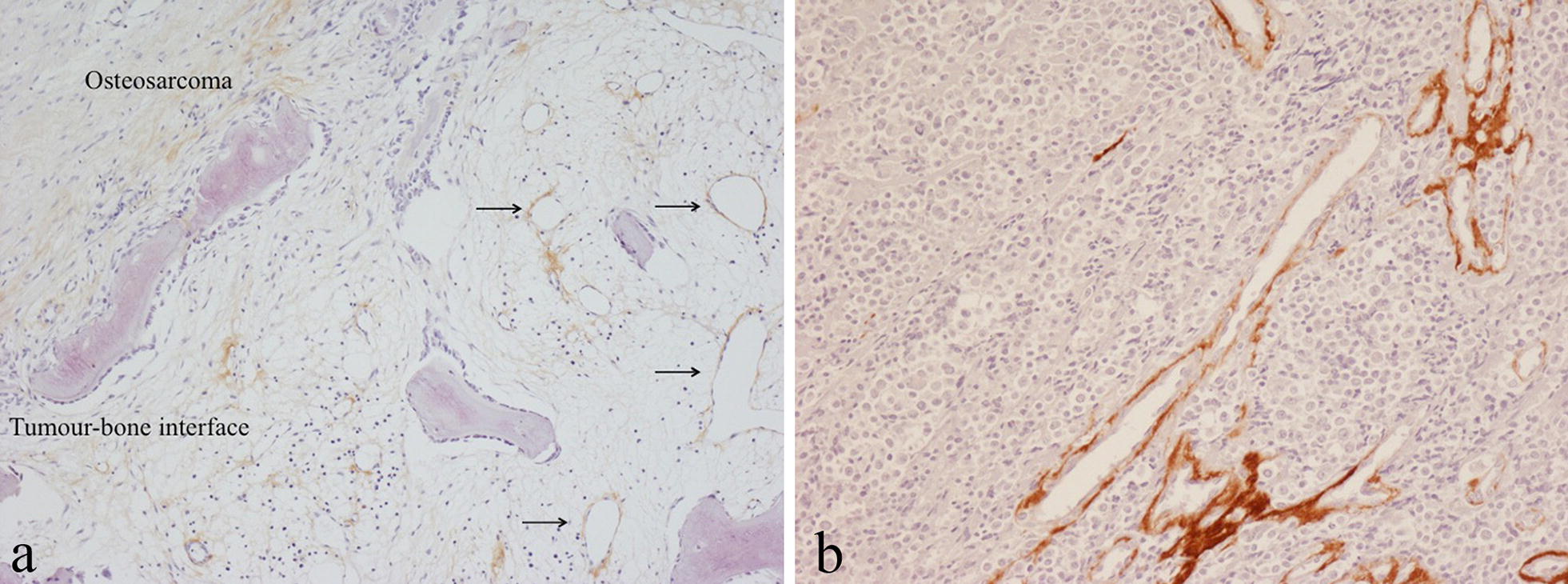
Immunohistochemical staining for periostin in: **a** osteosarcoma showing staining of vessels and matrix in fatty marrow in non-neoplastic bone at the tumour margin (arrowed); **b** metastatic melanoma showing prominent staining of vessels within the tumour

Staining for periostin was noted in non-neoplastic bone at the edge of growing benign and malignant primary bone tumours (Fig. 4a); this was in areas of fatty marrow in which there was fibrosis, increased vascularity and reactive bone formation with prominent staining often noted in the smooth muscle wall of small blood vessels with lining endothelial cells unstained. Infiltrating secondary carcinomas and melanomas that had metastasised to bone showed a similar pattern of staining for periostin in surrounding non-neoplastic bone. Strong periostin staining of vessels was also seen within metastatic tumours (Fig. 4b).

### Periostin expression in OA and RA

**Fig. 5:**
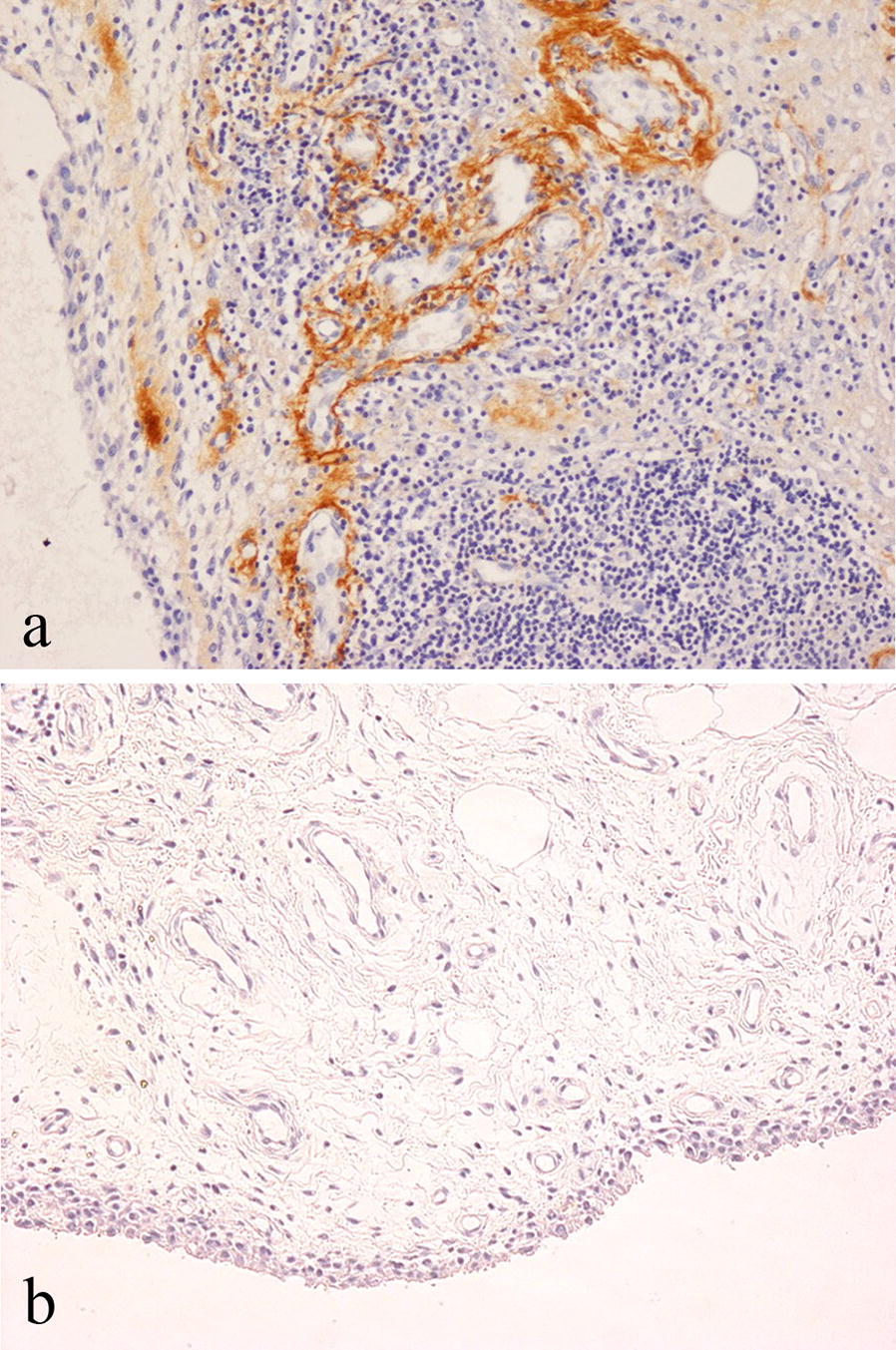
Immunohistochemical staining for periostin in: **a** RA synovium showing prominent staining of vessels in the subintima; **b** OA synovium

**Fig. 6:**
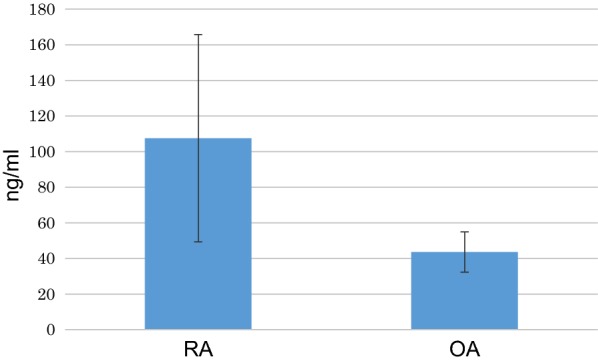
Amount of periostin in synovial fluid samples from RA and OA patients (n = 9), quantified using ELISA

Immunohistochemistry showed no staining for periostin of synovial lining cells in OA or RA synovium. Strong staining for periostin was noted in RA in the superficial subintima where there was staining of the fibrous tissue matrix and the smooth muscle wall of small blood vessels in areas of increased vascularity (Fig. 5a); in contrast, non-inflammatory OA synovium showed no subintimal periostin staining; (Fig. 5b). Periostin was also expressed in the matrix around large vessels in the deep subintima and capsule of RA joints. ELISA studies showed that the average human periostin concentration in synovial fluid was 107.4 ng/ml and 67.1 ng/ml in RA patients and OA patients respectively (Fig. 6). This was not of statistical significance (p = 0.29), but higher periostin levels were more frequently seen in RA than OA synovial fluid.

## Discussion

In this study, we have characterised periostin expression in neoplastic and non-neoplastic lesions of bone and joint. In keeping with its role in bone matrix formation, periostin was found to be strongly expressed in osteoid/bone-forming lesions; it was also noted in the mineralised chondroid/osteoid matrix of chondroblastomas and clear cell chondrosarcomas and in the connective tissue matrix of other primary bone tumours. Periostin expression was also prominent in areas of reactive host bone around infiltrating primary and secondary bone tumours, suggesting a role for this matricellular protein in tumour progression.

Periostin is a 90-kDa secreted protein which binds to type I collagen and other ECM proteins including fibronectin, Notch1, tenascin-C and BMP-1 [3–6, 18–20]; periostin acts to increase osteoblast proliferation, differentiation, adhesion and survival, and plays a key role in bone matrix formation. Periostin plays a role in bone remodelling by regulating collagen cross-linking and fibrillogenesis. In periostin-null mice, collagen fibrillogenesis is disrupted in the periosteum and mechanical loading results in a disorganised matrix formation. In addition, periostin expression is associated with reduced sclerostin and preservation of bone mass. Periostin increases ECM production by fibroblasts/myofibroblasts and promotes mesenchymal stem cell differentiation into osteoblasts, resulting in the formation of bone matrix. Periostin is known to function as a signalling molecule through integrin receptors and WNT-β-catenin pathways whereby it stimulates osteoblast function and bone formation.

Periostin was originally called osteoblast specific factor-2 and in this study we have shown that periostin is highly expressed in reparative lesions associated with osteoid/woven bone formation, such as fracture callus and myositis ossificans as well as in benign and malignant bone-forming tumours. Expression of periostin in osteosarcoma has previously been reported with high expression being correlated with tumour angiogenesis and poor prognosis [14, 15]. We noted periostin expression in both low-grade parosteal osteosarcomas and high-grade conventional osteosarcomas as well as in lung metastases of osteosarcoma with the extent of periostin expression appearing to be more related to formation of an osteoid matrix than histological parameters of tumour grade. Strong periostin expression was consistently noted in fibrous dysplasia and osteofibrous dysplasia, fibro-osseous bone tumours in which there is formation of woven bone with intramembranous ossification similar to that which occurs beneath the periosteum. Periostin was also seen in cellular fibrous tissue and areas of reactive osteoid/bone formation in other bone lesions, including simple bone cyst, ABC, fracture callus and myositis ossificans. It was also noted in the connective tissue matrix around mononuclear cells in giant cell tumour of bone; these cells are known to exhibit several osteoblast markers including alkaline phosphatase, RUNX2, osterix and RANKL [21]. Focal but less prominent staining for periostin was also noted in cellular and collagenous fibrous tissue of other primary bone tumours including non-ossifying fibroma and undifferentiated pleomorphic sarcoma.

Periostin was strongly expressed in the perichondrium covering osteochondromas and in areas of endochondral ossification at the base of growing osteochondromas but there was no staining in cells or matrix of the cartilage cap. Periostin expression was absent in other cartilage tumours including enchondroma and low/high-grade conventional chondrosarcoma. Our findings contrast with those of Lai and Chen [22], who identified periostin in chondrosarcoma and enchondroma using a commercial mouse monoclonal antibody TA804575 (OriGene Technologies, Inc., Rockville, MD, USA); in our study we employed a rabbit polyclonal antibody that had been characterised in previous investigations [13, 17]. In both chondroblastoma and clear cell chondrosarcoma, strong expression of periostin was noted in areas of chondroid matrix formation. In chondroblastoma, the chondroid matrix has been described as “chondrosteoid” by some observers [23]; it has been shown that this matrix contains dentine-matrix protein-1 and sclerostin, proteins found in newly formed osteoid [24, 25]. Clear cell chondrosarcoma, which is considered by some observers to be a malignant form of chondroblastoma [23], also showed expression of periostin in areas of woven bone formed within the clear cell cartilaginous stroma.

We consistently noted increased expression of periostin in non-lesional bone at the edge of growing benign and malignant bone tumours. It has been shown that periostin plays a role in the progression of inflammatory and neoplastic lesions [6, 11, 12, 23]. Periostin is known to be expressed by fibroblasts in RA, carcinomas and other malignant tumours [26–32]. Periostin is known to stimulate cell/matrix adhesion and migration of the endothelial cells through interaction with αVβ3 [33]. Endothelial cells strongly express αVβ3 when stimulated by growth factors produced in inflammation, wound healing and tumours. We noted that periostin was frequently expressed in the smooth muscle wall of small blood vessels within non-lesional bone around growing tumours, both benign and malignant. Interactions between periostin and vascular endothelial growth factor (VEGF) and its receptors are thought to play a key role in physiological and pathological angiogenesis [6, 12, 29, 34–36]. Periostin is strongly expressed by vascular smooth muscle cells, particularly those which are activated and migrating from the media to the intima or proliferating and synthesising matrix proteins. It has been shown that in breast carcinoma, squamous cell carcinoma and other tumours, blood vessel density in periostin-positive tumours is higher than in periostin-negative tumours with increased tumour invasion and metastasis being reported in these periostin over-expressing tumours [28, 37–40]. We noted prominent staining of the smooth muscle wall of small blood vessels in malignant tumours that had metastasised to bone.

There were similarities in the pattern of periostin expression in inflamed RA synovium and growing bone tumours. Periostin is known to be involved in the migration of synovial fibroblasts associated with RA pannus formation and joint destruction [4, 10]. We noted strong expression of periostin in the subintimal connective tissue matrix and smooth muscle wall of small blood vessels in RA synovium, indicating that periostin-associated angiogenesis may play a role in RA disease progression. We also noted higher levels of periostin in RA compared with OA synovial fluid and little staining for periostin in OA synovium. Increased levels of periostin have been associated with tumour angiogenesis, metastatic potential and poor prognosis in osteosarcoma patients [14, 15]. It has been shown that small interfering RNA against periostin significantly reduces the migration of fibroblast-like cells in RA [10]. Analogously, inhibition of periostin gene expression suppresses the proliferation and invasion of U2OS osteosarcoma cells [41] Our immunohistochemical finding of increased expression of periostin at the edge of growing bone tumours is in keeping with a role for periostin in tumour growth and, taken with the results of previous studies, suggests that periostin could represent a potential therapeutic target to control the growth of osteosarcoma and other bone tumours.

## Conclusions

In keeping with its known role in modulating the synthesis of collagen and other extracellular matrix proteins in bone, strong periostin expression was noted in benign and malignant lesions forming an osteoid or osteoid-like matrix. Periostin was also noted in other bone tumours and was found in areas of reactive bone and increased vascularity at the edge of growing tumours, consistent with its involvement in tissue remodelling and angiogenesis associated with tumour progression.

## Abbreviations

ABC: aneurysmal bone cyst
BMP-1: bone morphogenetic protein-1
ECM: extracellular matrix
ELISA: enzyme linked immunosorbent assay
GCTB: giant cell tumour of bone
OA: osteoarthritis
RA: rheumatoid arthritis
RANKL: receptor activator of nuclear factor kappa-B ligand
RUNX2: Runt-related transcription factor 2
SF: synovial Fluid
VEGF: vascular endothelial growth factor
